# Influences on Patient Uptake of and Engagement With the National Health Service Digital Diabetes Prevention Programme: Qualitative Interview Study

**DOI:** 10.2196/40961

**Published:** 2023-02-28

**Authors:** Jamie Ross, Sarah Cotterill, Peter Bower, Elizabeth Murray

**Affiliations:** 1 Centre for Primary Care Wolfson Institute of Population Health Science, Barts and The London School of Medicine and Dentistry Queen Mary University of London London United Kingdom; 2 Division of Population Health Health Services Research & Primary Care University of Manchester Manchester United Kingdom; 3 E-health Unit Department of Primary Care and Population Health University College London London United Kingdom

**Keywords:** diabetes prevention, digital health interventions, engagement, qualitative research, mobile phone

## Abstract

**Background:**

Digital diabetes prevention programs (digital-DPPs) are being implemented as population-based approaches to type 2 diabetes mellitus prevention in several countries to address problems with the uptake of traditional face-to-face diabetes prevention programs. However, assessments of digital-DPPs have largely focused on clinical outcomes and usability among those who have taken them up, whereas crucial information on decision-making about uptake (eg, whether a user downloads and registers on an app) and engagement (eg, the extent of use of an app or its components over time) is limited. Greater understanding of factors that influence uptake and engagement decisions may support large-scale deployments of digital-DPPs in real-world settings.

**Objective:**

This study aimed to explore the key influences on uptake and engagement decisions of individuals who were offered the National Health Service *Healthier You*: Digital Diabetes Prevention Programme (NHS-digital-DPP).

**Methods:**

A qualitative interview study was conducted using semistructured interviews. Participants were adults, aged ≥18 years, diagnosed with nondiabetic hyperglycemia, and those who had been offered the NHS-digital-DPP. Recruitment was conducted via 4 providers of the NHS-digital-DPP and 3 primary care practices in England. Interviews were conducted remotely and were guided by a theoretically informed topic guide. Analysis of interviews was conducted using an inductive thematic analysis approach.

**Results:**

Interviews were conducted with 32 participants who had either accepted or declined the NHS-digital-DPP. In total, 7 overarching themes were identified as important factors in both decisions to take up and to engage with the NHS-digital-DPP. These were knowledge and understanding, referral process, self-efficacy, self-identity, motivation and support, advantages of digital service, and reflexive monitoring. Perceptions of accessibility and convenience of the NHS-digital-DPP were particularly important for uptake, and barriers in terms of the referral process and health care professionals’ engagement were reported. Specific digital features including health coaches and monitoring tools were important for engagement.

**Conclusions:**

This study adds to the literature on factors that influence the uptake of and engagement with digital-DPPs and suggests that digital-DPPs can overcome many barriers to the uptake of face-to-face diabetes prevention programs in supporting lifestyle changes aimed at diabetes prevention.

## Introduction

### Diabetes Prevention

Diabetes is a global health priority. The World Health Organization estimates that diabetes was the seventh leading cause of death across the world in 2016 [1]. In the United Kingdom, approximately 3.9 million people are diagnosed with diabetes, of which 90% are diagnosed with type 2 diabetes mellitus (T2DM) [2], and a further 5 million people are estimated to have nondiabetic hyperglycemia (raised blood glucose levels or prediabetes) in England [3]. T2DM is associated with obesity, lack of physical activity, and genetic risk factors such as ethnicity [4]. For many people, T2DM may be preventable by changes to diet and activity [5,6]. There is high-quality international evidence that intensive group-based programs focusing on healthy eating, weight loss, and increased exercise can reduce the risk of progression to T2DM in people with prediabetes [7-10].

In 2016, the National Health Service *Healthier You*: Diabetes Prevention Programme (NHS-DPP) was established with the aim to prevent or delay the onset of T2DM in adults in England who are identified to be at high risk. The NHS-DPP delivers behavioral interventions that encourage increased physical activity and a healthy diet, in addition to weight loss, for people who are overweight. The face-to-face version of the NHS-DPP (NHS-f2f-DPP) involves individuals attending at least 13 in-person group‐based sessions over a period of at least 9 months. Early outcome data indicate that the NHS-f2f-DPP led to weight loss and glycated hemoglobin reductions among individuals who completed the program [9,11], and it is associated with reduced population incidence of T2DM [12].

However, face-to-face diabetes prevention programs (f2f-DPPs) do not suit everybody, and there are recognized barriers to attendance [13]. People who work or have caring responsibilities may find it difficult to attend in-person programs [14]. Furthermore, f2f-DPPs are usually delivered in groups, meaning that those who do not like groups may find it difficult to participate [15]. Only 56% of those referred to the NHS-f2f-DPP during the first 12 months of the program took up the referral [16]. To expand the reach and access of DPPs, digital diabetes prevention programs (digital-DPPs) have been suggested as complementary alternatives to f2f-DPPs [13].

### Digital Health Interventions for Diabetes Prevention

Digital health interventions (DHIs) have been shown to be effective in increasing physical activity, changing diets, and promoting weight loss in general populations [17-19]. DHIs offer many benefits, for example, being able to integrate principles of *persuasive design* such as personalization, gamification, and social influence and behavior change techniques such as self-monitoring to encourage users to take up behavior change [20]. They can also capitalize on habitual smartphone and internet use among the general population to deliver intense behavior change support programs that are highly scalable [21].

There is emerging evidence to suggest that DPPs can be delivered effectively using digital technologies and achieve outcomes comparable with f2f-DPP in those who take them up [21-25]. In addition, digital-DPPs may be more acceptable to some people than f2f-DPPs, as they may be easier to fit into busy lifestyles, avoid the perceived stigma associated with attending a group, and have the potential for tailoring and personalization [15,26]. However, little is understood about how to best translate digital health solutions into real-world conditions and ways that engage and meet the needs of diverse stakeholders [20].

Early analyses of the NHS-DPP showed that the uptake was significantly lower for those of working age [27]. To address inequalities in access according to age, a digital pathway was introduced in 2019 [28]. This digital version of the NHS-DPP uses DHIs including apps that allow users to access health coaches and set and monitor goals electronically and access educational material and peer support groups and wearable technologies that monitor levels of physical activity.

### Uptake of and Engagement With Digital-DPPs

Uptake and engagement with DHIs are generally agreed to be prerequisites for effectiveness. Low rates of uptake, retention, and program completion represent a major barrier to effective implementation and public health impact of digital interventions [29-33], and a greater understanding of why people take up and engage with digital-DPPs is important in promoting their widespread impact.

A recent study of factors influencing decisions to attend the NHS-f2f-DPP identified knowledge and understanding of T2DM, perceptions about illness, and social support as important factors in decisions to attend [34]. A recent synthesis of qualitative studies of barriers to and facilitators of lifestyle change in people with prediabetes identified perceptions of the importance of initiating lifestyle change, strategies, and coping mechanisms for maintaining lifestyle changes and supportive relationships and environments as important [35]. Studies on attendance at other services to which individuals are referred via primary care, including diabetes self-management education [15] and weight management services [36], highlight the importance of the way in which referrals are made by health care professionals (HCPs) in encouraging attendance.

There may be additional and unique factors that influence decisions to take up digital-DPPs including digital health literacy [37], technological self-efficacy, and perceived usefulness [38,39]. A recent review of the factors that promote adherence (defined by the authors as “the degree to which the user followed the program as it was designed”) to mobile health apps for a range of health conditions found intervention and patient-related factors to be important. User-friendliness, technically stable app design, customizable push notifications, personalized app content, and passive data tracking were some of the app features that influence adherence. Furthermore, certain user characteristics were associated with low adherence including lack of technical competence, low health literacy, low self-efficacy, and low education level [40]. However, to the best of our knowledge, no study has explored uptake and engagement decisions regarding DHIs in the context of diabetes prevention.

### Goals of This Study

This study aimed to explore the key influences on participants’ decisions to take up and engage with the National Health Service *Healthier You*: Digital Diabetes Prevention Programme (NHS-digital-DPP).

## Methods

### Design

Qualitative study using semistructured interviews was conducted with individuals who were offered the NHS-DPP with a choice of face-to-face or digital delivery.

### Participants

Individuals eligible for the NHS-DPP are adults aged ≥18 years and diagnosed with nondiabetic hyperglycemia. This is defined as having at least one glycated hemoglobin reading of 42 to 47 mmol/mol or at least one fasting blood glucose reading of 5.5 to 6.9 mmol/L in the 24 months before referral. People already diagnosed with diabetes and pregnant women are not eligible for the program.

In most areas, those eligible to participate in the NHS-DPP were identified from primary care lists or during NHS Health Checks. NHS Health Checks are offered to people aged between 40 and 74 years living in England, who have not previously been diagnosed with certain conditions [41]. Participants were informed that they are at high risk of developing T2DM and offered referral to the NHS-DPP.

The COVID-19 pandemic had 2 main impacts on the delivery of the NHS-DPP. First, from March 2020, the NHS-f2f-DPP option was not available, and instead, a *remote group* option was established, which comprised group sessions conducted via a web-based platform or telephone. Second, in August 2020, the NHS-DPP expanded access by including a self-referral route via a web-based risk tool. Eligibility to self-refer to the program was based on the Diabetes UK risk tool (a validated T2DM risk assessment tool) completed via the Diabetes UK website. Those scoring at or above a risk threshold were guided to self-refer with their local NHS-DPP provider (identified by postcode).

Thus, during the time this study was conducted (October 2021 to March 2022), individuals could access the NHS-DPP via referral from primary care or via the self-referral route. Those who took up the referral were offered a choice of delivery mode, which, during the study, was remote groups or the NHS-digital-DPP. There was also the option to defer until a later date.

### Intervention

The NHS-digital-DPP is delivered by 4 independent providers commissioned to deliver the 9-month behavior change program. Participants were offered 1 of the 4 service providers’ digital programs, depending on which provider was commissioned to deliver the digital service in their local geographical area. Although based on a common NHS England service specification [28,42], the DHIs vary in terms of their provision of materials, inclusion of wearables (eg, accelerometers and wireless weighing scales), extent of human support provided (ranging from a brief onboarding phone call to weekly coaching phone calls), delivery platform (smartphone app and website), and amount and format of educational materials (websites, emails, etc). Table 1 provides a summary of the providers’ features.

**Table 1:** Features of the NHS-digital-DPP^a^ provider programs.

NHS-digital-DPP provider’s features	Provider A	Provider B	Provider C	Provider D
Materials provided to service user	Program app	Program app and program handbook	Program app	Program app, program handbook, recipe book, wireless scales, and activity tracker
Educational content	42 web-based articles	Weekly web-based articles	Bite-sized videos and written modules to supplement participant learnings—these are assigned by the health coach	Web-based articles that are unlocked daily and 8 optional 4-week web-based courses
Professional input	Health coaching via series of scheduled telephone calls and web-based chat	Access to health coaches via chat function	Health coaching via initial telephone call, then regular video messages and web-based chat	Health coaching in a web-based message service with a group of approximately 10 people (access to health coach in group or one-on-one chat)
Peer support	Not part of service at time of study	Optional web-based discussion forum	Optional web-based discussion forum	Optional web-based discussion forum

^a^NHS-digital-DPP: National Health Service *Healthier You*: Digital Diabetes Prevention Programme.

### Study Sampling and Recruitment

Sampling aimed to recruit a maximum variation sample of patients eligible for the NHS-DPP (refer to the previous sections), selected to vary by age, sex, ethnicity, socioeconomic status, geographical area, NHS-digital-DPP provider, and level of engagement with the NHS-digital-DPP. Participants were recruited via the 4 providers of the NHS-digital-DPP and via 3 primary care practices located within one clinical commissioning group in North London between October 2021 and March 2022.

Providers contacted 2 groups of participants: those who had taken up the NHS-digital-DPP and those who had declined the NHS-digital-DPP in favor of participating in the remote group delivery mode. As the focus of the study was on decision-making around uptake, sampling was targeted at those who had recently been offered the NHS-digital-DPP. Therefore, on a monthly basis, providers emailed anyone referred in the previous 4 weeks.

The aim of the additional recruitment via primary care practices was to capture participants who had been invited to participate in the NHS-digital-DPP but had not taken up the offer. A clinical commissioning group, which had in place a locally enhanced service for prediabetes monitoring, meaning referrals to the NHS-DPP and patient responses to referrals were routinely recorded, and which was only offering the remote or NHS-digital-DPP, was selected. Emails and letters were sent in monthly batches via the practices to any patient with a record of having declined or not responded to an invitation during the recruitment period (October 2021 to March 2022). Interested participants (from both recruitment routes) were asked to contact the research team, who then sent study documents and organized a time for interview.

To recruit a maximum variation sample, researchers worked with the local National Institute for Health and Care Research Clinical Research Network to identify and sample primary care practices based on a range of factors, including deprivation scores, ethnic diversity, and diabetes prevalence. Researchers also worked iteratively with providers to increase the representation of participants from ethnic minority backgrounds and regions of high deprivation. At several time points, providers were asked to target recruitment emails to participants from those groups specifically.

In total, 2051 participants were contacted by the NHS-digital-DPP providers, and 35 were contacted by primary care practices. In total, 3.17% (65/2051) initial expressions of interest were received from the provider recruitment and 11% (4/35) were received from primary care. Recruitment from providers ceased when data saturation had been reached, which was deemed to happen at interview 28, when no new themes were emerging. Interviews were conducted with all respondents (4/4, 100%) recruited via primary care. Despite considerable efforts to recruit those who had not taken up the NHS-digital-DPP, this proved to be challenging, and recruitment was stopped for this group at the end of the study data collection period. At this point, it is believed that data saturation had not been reached (refer to the *Discussion* section).

### Ethics Approval and Informed Consent

Ethics approval for the study was obtained from the North West–Greater Manchester East National Health Service (NHS) research ethics committee (17/NW/0426). Verbal informed consent was recorded via an audio recorder for each participant before participation.

### Data Collection

Topic guides were developed based on constructs from the health belief model (HBM) [43]. The HBM is one of the most widely used conceptual frameworks for explaining and changing individual health behavior and posits that individuals’ perceived susceptibility to and severity of a disease influence the perceived threat of the disease, which predicts the likelihood of self-management behaviors [44]. Furthermore, for people to comply with participatory preventive interventions, they need to perceive both the risk of the condition in question and the potential benefit of the intervention. The HBM has been widely applied to studies on prediabetes [45] and the development and evaluation of DHIs [46]. The topic guide included the following 6 domains of the HBM: perceived susceptibility, perceived severity, perceived beneﬁts, perceived barriers, self-eﬃcacy, and cues to action (Multimedia Appendix 1). For example, to examine the perceived susceptibility, we asked the following question: “How likely do you feel it is that you will develop diabetes?” The topic guide also included specific questions related to experiences with the NHS-DPP, and a separate set of questions was asked to those who had taken up and those who had not taken up the NHS-DPP. Topic guides were developed iteratively throughout the study to explore emerging areas of interest. All interviews were semistructured and conducted by the same researcher (JR). Owing to COVID-19–related restrictions, participants were given a choice between phone or Zoom (Zoom Video Communications Inc) interviews. Most participants (26/32, 81%) opted for Zoom. Interviews were recorded using an audio recorder. Demographic data were collected from participants via a proforma at the end of each interview. Audio recordings were securely transferred to a transcription company for transcription. After transcription, the researcher checked the transcripts for accuracy against the audio recordings and anonymized the transcripts.

### Data Analysis

Data collection and analysis were conducted concurrently. Anonymized transcripts were coded using NVivo (version 10; QSR International) software. The analysis was conducted using an inductive thematic analysis approach [47]. This involved 6 phases: data familiarization, coding, identification of candidate themes, review and revision of themes, definition and naming of themes, and analysis and interpretation of patterns across the data. Constant comparative analysis was conducted by reviewing the transcripts and exploring the identified themes in subsequent interviews until data saturation was achieved. Codes and emerging themes were generated by the lead researcher and discussed within the multidisciplinary team to promote rigor and transparency in the analysis. The generated themes were considered through the lens of HBM to aid the interpretation of findings.

### Patient and Public Involvement

The study was guided by an expert advisory group comprising 5 people with lived experience of prediabetes. The study was discussed with this group, which provided input (written and verbal) into a draft of the topic guide, particularly focusing on the relevance, importance, clarity, and wording of the questions. Changes to the topic guide suggested by the patient and public involvement group were implemented. Emerging findings were presented to the group that commented on preliminary themes. Ongoing work with the group includes coproducing a video to disseminate the research findings.

## Results

### Characteristics of Study Participants

In total, 32 interviews were conducted, each lasting 42 minutes on average. Demographic characteristics are summarized in Table 2. Of the 32 participants, 24 (75%) had taken up the offer of the NHS-digital-DPP, 4 (13%) had declined the offer in favor of remote group delivery, and 4 (13%) had been offered participation but had not taken up the offer. It is worth noting that in this study, all remote group participants (4/4, 100%) were from provider B, which gave participants access to their DHI and web-based group meetings.

Of the 28 participants who took up the NHS-DPP, 22 (79%) had been referred directly by their primary care practice, 2 (7%) had been informed about the NHS-DPP by primary care practices but were not provided with a referral and contacted providers themselves, and 4 (14%) had not been informed about the NHS-DPP by their primary care practice and had instead used the web-based risk tool and self-referred. Of the 24 participants who had taken up the NHS-digital-DPP, most (n=19, 79%) participants reported daily use; however, 21% (5/24) of the participants reported less frequent use.

Compared with the 3623 participants in a pilot study of the NHS-digital-DPP [24], the participants had the same mean age (58, SD 10.7 years), had similar proportion of men and women (men: 50% vs 49%), and were equally ethnically diverse (White British: 68% vs 68%). They had more years of education (higher education: 78% vs 29%) and were from more affluent areas (from least deprived areas: 25% vs 15%).

**Table 2:** Participant characteristics.

Characteristics					Values
**All participants (N=32)**					
	Age (years), mean (SD)			58.3 (10.7)	
		**Sex, n (%)**			
			Female	16 (50)	
			Male	16 (50)	
		**Ethnicity, n (%)**			
			White	22 (69)	
			Mixed	2 (6)	
			Black	2 (6)	
			Asian	2 (6)	
			Other	4 (13)	
		**Education, n (%)**			
			None	1 (3)	
			Secondary school education (GCSE^a^, O level^b^, or CSE^c^)	6 (19)	
			Higher	25 (78)	
		**Self-rated computer skills, n (%)**			
			Basic	6 (19)	
			Intermediate	13 (41)	
			Advanced	13 (41)	
		**Home internet access, n (%)**			
			Yes	32 (100)	
		**Owns smartphone, n (%)**			
			Yes	31 (97)	
			No	1 (3)	
		**Perceptions of current health, n (%)**			
			Excellent	2 (6)	
			Very good	10 (31)	
			Good	12 (38)	
			Fair	5 (16)	
			Poor	3 (9)	
		**Current occupational status, n (%)**			
			Paid work	20 (63)	
			Unemployed	2 (6)	
			Voluntary work	1 (3)	
			Not working owing to disability or ill health	1 (3)	
			Retired	8 (25)	
		**Known someone with T2DM^d^, n (%)**			
			Yes	26 (81)	
			No	6 (19)	
		**Parent or sibling with T2DM, n (%)**			
			Yes	15 (47)	
			No	17 (53)	
		**Live with others, n (%)**			
			Yes	25 (78)	
			No	7 (22)	
		**Deprivation, n (%)**			
			IMD^e^ 1 (most deprived)	4 (13)	
			IMD 2	7 (22)	
			IMD 3	6 (19)	
			IMD 4	7 (22)	
			IMD 5 (least deprived)	8 (25)	
		**Referral offer source, n (%)**			
			Primary care	26 (81)	
			Self-referral	6 (19)	
	**Offer, n (%)**				
			Accepted	24 (75)	
			Declined in favor of remote group	4 (13)	
			Not taken up	4 (13)	
**Digital NHS-DPP^f^ group only (n=24)**					
	**Use of NHS-digital-DPP^g^ DHIs^h^, n (%)**				
			Daily	19 (79)	
			Several times a week	2 (8)	
			Once a week	2 (8)	
			Less than once a week	1 (4)	
	**NHS-digital-DPP provider, n (%)**				
			A	8 (33)	
			B	2 (8)^i^	
			C	8 (33)	
			D	6 (25)	
	**Time using DHI at the time of interview (weeks), n (%)**				
			1-2	1 (4)	
			2-3	5 (21)	
			3-4	9 (38)	
			4-5	9 (38)	

^a^GCSE: General Certificate of Secondary Education.

^b^O level: Ordinary level.

^c^CSE: Certificate of Secondary Education.

^d^T2DM: type 2 diabetes mellitus.

^e^IMD: Index of Multiple Deprivation (the official measure of relative deprivation for small areas in England).

^f^NHS-DPP: National Health Service *Healthier You*: Diabetes Prevention Programme.

^g^NHS-digital-DPP: National Health Service *Healthier You*: Digital Diabetes Prevention Programme.

^h^DHI: digital health intervention.

^i^The 17% (4/24) of participants who opted for remote groups were also from this provider.

### Factors Influencing the Decisions to Adopt and Engage

#### Overview

Overall, seven overarching themes were identified: (1) knowledge and understanding, (2) referral process, (3) self-efficacy, (4) self-identity, (5) motivation and support, (6) advantages of digital service and response efficacy, and (7) reflexive monitoring. Some themes encompassed factors that were related to uptake of and engagement with the NHS-DPP generally, whereas others were specific to the NHS-digital-DPP. These themes are summarized in Multimedia Appendix 2.

#### Knowledge and Understanding

This theme relates to participants’ knowledge and understanding about T2DM and NHS-DPP.

Participants reported mixed understanding about what prediabetes and T2DM are and how one relates to the other. Several participants had not heard of the term *prediabetes* before their diagnosis, and there was some confusion about what it meant. For others, especially those with a family history of diabetes, there was greater understanding about the nature of the condition. Participants described prediabetes in terms of “sugar levels higher than they should be” (participant 5), being “at risk from diabetes” (participant 9), and “borderline” (participants 4, 8, 11, 19, 22, and 28) for diabetes and understood that prediabetes could progress to “full-blown diabetes” (participant 19).

For many participants, the receipt of their prediabetes diagnosis was unexpected and was described as a “shock” (participants 1, 3, 10, 11, 21, 22, 27, and 30). This was particularly true in several cases where participants had not known they were being “tested” for diabetes risk or had not experienced any symptoms of ill health. The surprise at the diagnosis was more apparent for participants who did not identify as being a “typical” person with diabetes, who were described in terms of being overweight and having poor eating habits:

I was really very shocked because...this type of diabetes...to me...people that get that are a bit lazy and they don’t look after themselves.

Participant 22; female; aged 58 years; provider D

For some participants, the surprising nature of the diagnosis affected the readiness to take up the NHS-DPP. A person who declined the program reported feeling very overwhelmed by the diagnosis to take preventive action (participant 29). Another participant described that despite knowing about the potential serious consequences of diabetes, they were not currently in the right “headspace” (participant 32) to engage with making behavior changes or had other health concerns and prediabetes was not perceived as a priority. However, others described the diagnosis as a “wake up call” (participants 3, 21, and 22), which prompted a desire to become healthy:

One minute I was OK and the next I had all these things thrown at me and I just got panicky...I got scared at everything they were telling me.

Participant 29; female; aged 52 years; nonadopter

I put them off a couple of times and said, “I can’t deal with this at the moment”...it [cataract surgery] was taking up all my sort of energy and all my fretting. I didn’t have time to – I didn’t care about anything else.

Participant 16; female; aged 63 years; provider A

There were those who, despite being diagnosed with prediabetes, reported that they had not been provided with sufficient (or any) information about what this diagnosis meant. Several participants reported being informed about their risk of diabetes via a letter from their primary care practice and had not spoken to an HCP about it. Some participants commented on the perceived lack of importance given to the diagnosis by their HCP, which in turn influenced their perceptions of risk:

There was no urgency...because it wasn’t discussed in a manner that made it feel urgent, I didn’t treat it as urgent.

Participant 32; male; aged 50 years; nonadopter

Those who had less understanding about diabetes risk were either motivated to join the NHS-DPP to become more educated, or in contrast, reported less inclination to embark on changes to lifestyle behaviors immediately. For example, there was some confusion about disease progression, with some believing that T2DM was a future eventuality that should not cause immediate concern:

I was very happy with the offer. I wanted to do it straight away, because I didn’t know how to make any changes...And so any advice or help, I really, really needed...the guidance.

Participant 25; female; aged 57 years; provider C

We know that if I just carried on doing what I was doing that I could well go into type 1 diabetes. That, in itself, is not too much of a problem because you’ve got quite a way to go, I guess, until you get to type 2. So when you get near the value of type 2, then you’re really starting to say, “Well, I’ve got to really do something here.”

Participant 5; male; aged 75 years; remote group

#### Referral Process

This theme includes factors related to the implementation of the NHS-DPP, which affected the participants’ decisions to take it up.

There was a lack of information in the offer of the NHS-DPP from primary care. Many participants reported not being sure about what they may get from the program because this had not been made clear to them at the point of referral. There was a sense from participants that HCPs did not know much about what they were offering to patients:

She [nurse] didn’t know much about it. She just said, “I’ll put your name forward.” And I haven’t talked to her since then. It’s not at all what I expected or what I thought I was signing up for but then to be fair I didn’t know what I was signing up for.

Participant 15; female; aged 75 years; provider A

Most participants reported not knowing that a digital option for the NHS-DPP was available until they had an initial conversation with the program providers, at which point they were offered a choice of service. Overall, 6% (2/32) of the participants had declined the offer from their primary care practice as they felt that they were too busy to embark on a program to support lifestyle changes. When the digital version was discussed with these participants during our interviews, both of them suggested this would have been more appealing and likely taken up (participants 31 and 32):

What, do it online, is that what you’re saying...Yeah. I mean that would probably be easier I think.

Participant 31; male; aged 64 years; nonadopter

When the NHS-DPP was discussed in more detail with participants in primary care services, it positively affected their decision to take it up:

Yeah. She [nurse] said she’d directed a couple of people for their weight, and they were doing quite well, so I thought – I knew it was something that I was going to sign up, definitely, by the way she described it.

Participant 4; female; aged 39 years; provider B

In some cases, participants had not been informed about the NHS-DPP by primary care, and in other cases, although they were informed about their risk of T2DM from their primary care practice, no offer of participation in the NHS-DPP had been received. This prompted participants to search for information on their own. These participants reported finding about the NHS-DPP via Facebook advertisements, by Googling, and from the NHS website and had subsequently gained access by using the web-based risk score:

It was one of those – it was a form that you clicked on, and it was like assess your risk of diabetes in two minutes, and you click on this form, and it gives you some questions to answer and then it gives – it spits out your risk of diabetes at the end, and then it sort of recommends actions that you can take.

Participant 14; male; aged 69 years; provider D

The fact that the NHS-DPP was referred to by and affiliated with the NHS was of critical importance for perceptions of trustworthiness and credibility. This was important to participants who contrasted the program with other digital sources of information, which they had found hard to verify or assess as credible. The NHS affiliation also gave participants confidence that the program would be efficacious in bringing about the desired outcomes:

Oh, I mean, it gave it credibility. I would hope, and I trust that the NHS would check the outcomes, have some sort of monitoring system.

Participant 11; female; aged 60 years; provider C

Many participants were offered referral via a letter or SMS text message, which was easily dismissed and did not carry the same seriousness as a conversation with a health professional. A participant reported not accepting the offer immediately because of this reason, and instead, waiting another 6 months before joining:

I suppose if the doctor had phoned me at that point and said, “Look...there’s this programme that might be able to help,” then I would certainly have said yes.

Participant 1; female; aged 66 years; remote group

There was also no follow-up from primary care about whether participants had taken up the offer of the NHS-DPP, which contributed to a sense that this was not an important thing to engage with and led to delays with participants taking up the offer:

For 50% (2/4) of the participants who had not taken up the program, this was because they had no recollection of receiving a letter from their primary care practice and no follow-up meant that they had not had further opportunity to be referred:

If there was a follow-up call from the surgery to say, “Have you taken it up, because if not, we’ll pass it on to somebody else?” [I] would say, “ooh, I’d better do it.”

Participant 2; male; aged 65 years; provider B

Even after accepting the referral, there were reports of delays in participants accessing the NHS-digital-DPP. Participants reported being almost ready to “quit” (participant 13) and “left to flounder” (participant 1) without knowing what to do after receiving their diagnosis (participant 12) owing to delays in program providers making contact. There were examples of considerable effort by some participants to get onto the program, with repeated phone calls to providers:

At one point I thought, “OK, that's not going to happen. Nobody’s going to get in touch.” I think he [GP] done his job from his end- three months is just – I feel that is a long gap.

Participant 8; male; aged 45 years; provider A

#### Self-efficacy

An important factor affecting self-efficacy (confidence in one’s ability) for diabetes prevention was the participants’ previous efforts to modify behavior, especially around changing diets and weight loss. Some participants reported having made considerable improvements to their diet and exercise behaviors after receiving the diagnosis of prediabetes and before embarking on the NHS-digital-DPP and thus felt confident that they would be able to keep this up and that an intervention to support this would be useful. However, others reported having previously failed with weight loss efforts or found certain aspects of behavior change to be difficult, and therefore, they were less confident that they would be able to make any sustainable changes:

I’ve done the quick fix diet, so I know I can do that now when I need to. If I need to lose a stone rapidly I can do that for a month and I’ll lose a stone.

Participant 16; female; aged 63 years; provider A

I’ll probably have four weeks where I’ve been quite well-behaved, and then it’ll just go off the rails again.

Participant 26; male; aged 59 years; provider C

Others, especially those who had family members with T2DM, drew on the genetic nature of risk to explain why they felt less capable of preventing disease progression, explaining that lifestyle modifications would not be sufficient to reduce their risk of developing T2DM. Thus, they were hesitant about how useful the NHS-DPP would be:

I didn’t feel that after a year of careful eating, I deserved to be pre-diabetic. And I’m still not convinced that I will be able to stop it. I kind of think my sister eats more healthily than me, I believe, and she managed to get in that category.

Participant 1; female; aged 66 years; remote group

After participants had embarked on using the program, many reported increased feelings of self-efficacy toward being able to make lifestyle changes and reduce the risk of developing diabetes. Increased self-efficacy was related to increased knowledge and was bolstered by seeing changes related to behavioral modifications such as weight loss:

But I feel fairly confident that now that I know what’s happening, so I can do something about it, whereas before, I didn’t know.

Participant 5; male; aged 75 years; remote group

#### Self-identity

A considerable barrier for some in deciding to embark on the program was that they did not identify as being the target population for an intervention they perceived as being primarily for weight loss:

The problem is, as I say, I’m not particularly typical. I’m not overweight, and whatever, and I eat – I’m not – I don’t eat perfectly. But my diet is not that bad.

Participant 27; female; aged 65 years; provider C

Other participants did not identify as the target users, as they perceived themselves to already have a good level of knowledge about healthy eating. Many participants questioned whether the NHS-digital-DPP would be of any benefit to them, as they considered themselves to not be in need of the “basic information” (participant 11) that they perceived the program would offer. Again, participants contrasted themselves with the type of person they perceived to need support in terms of knowledge:

I’d like to think I’m a semi-intelligent individual who understands a little bit about what we should and shouldn’t be eating and exercise and stuff and all that. So I wasn’t so sure that I was going to learn a great deal.

Participant 12; male; aged 53 years; provider A

Another subgroup of participants whose self-identity led them to question the usefulness of the NHS-digital-DPP to support them with lifestyle changes included those who had comorbidities and mobility issues. These participants perceived themselves as having needs that are different from those of other users and reported needing a service that was much more tailored to their individual needs:

Quite early on I did read that it would be tailored, that it would be a support programme for you. And then when I started it and I found that this felt very far from tailored or a support for me.

Participant 15; female; aged 75 years; provider A

#### Motivation and Support

The digital delivery particularly appealed to those who described themselves as being “self-motivated” (participant 12) to take action to change behaviors, perceiving the DHI as a facilitatory tool rather than as a main driver of change. This was often contrasted with the perception of face-to-face support, which was viewed as better for those who needed external motivation to change. Several people who had opted for digital delivery described not wanting to engage in group sessions as they were perceived to be aimed at people who needed more encouragement, who would not be able to achieve desired outcomes on their own with the support of just the DHI:

There are those sort of people that need that group encouragement, or outside motivation “You’re doing well, just adjust this,” a mentor, a support, I don’t think they would get on quite so well with the app.

Participant 12; male; aged 53 years; provider A

For those who had access to health coaches, they were perceived positively, and they influenced decisions to take up and engage. Many participants viewed health coaches as a way to instill accountability for their lifestyle changes. Some spoke about having sufficient knowledge to make changes to their lifestyles, but that staying motivated was more difficult. One of the main benefits of the health coaches was to help participants stay “on track” (participant 3) and accountable:

It was like “great, there’ll be someone that I can be accountable to,” and it was the accountability that attracted me to it with what I thought would be some good advice.

Participant 9; female; aged 53 years; provider A

However, some participants expressed disappointment regarding the lack of support from the DHIs. Participants discussed feeling demotivated to continue use because of lack of perceived support. This was particularly true for those using the DHI, which did not provide formal health coach support (provider B). Those who had not yet been contacted by the health coaches also reported feeling demotivated after joining. In some cases, participants reported waiting for weeks before having their first session with a health coach. Without access to a health coach, several participants reported feeling that all they were doing was tracking their food, and to make meaningful behavior changes, they required more support and a more structured approach to lifestyle changes:

And this is just literally logging. I don’t feel any kind of support or any benefit, you know.

Participant 13; female; aged 55 years; provider A

For those who opted for the remote group version, peer support was an important factor in this decision. These participants discussed wanting to feel as part of a community and to be able to draw on others’ experiences and share ideas:

It’s the chance to share experiences, successes and failures, when you talk through –when people have said, “Oh, I’ve had a really bad week...” it makes you sort of feel it’s not just me that does that...But also when people have had a success, or they’ve done something good...it sort of motivates you a little bit more.

Participant 3; female; aged 57 years; remote group

Although all the DHIs had an aspect of peer support, few participants reported engaging with these features, which included group messaging, chat rooms, and forums. Several participants stated that one of the main reasons for selecting digital was for the avoidance of having to interact with others, which they likened to self-help groups. This was consistent with the previously mentioned identities of these participants as not being “typical” of the prediabetic population:

I don’t really like the group aspect of it...in a group of people that are overweight and need to change...that group aspect of turning up, weighing in, all that, “Oh, aren’t you doing well?” I’m not really positive about that at all.

Participant 2; male; aged 65 years; provider B

For the few participants who reported having engaged with peer support features, there was some disappointment regarding the lack of group interactions, and others reported that they did not engage with these features because others were not engaging, suggesting that a critical mass of users had not been reached:

I did wonder whether there might be a kind of a bit of a group dynamic. And maybe this is where, if you were in a room with other people, that would develop, but it hasn’t, doesn’t seem to have anyway.

Participant 21; male; aged 43 years; provider D

#### Advantages of Digital Service

Perceptions of acceptability were pivotal in making decisions to engage with the NHS-digital-DPP. Many benefits were discussed in contrast to face-to-face and remote group delivery, particularly around the benefits of the convenience that a digital service could offer. The digital service particularly appealed to those who worked and those who had a dislike for groups:

Groups, which because of my mental health, I was really nervous about, and I probably wouldn’t have partook. But the fact that it was available in a digital form was – it just made us take the plunge to just try and go ahead and do it.

Participant 4; female; aged 39 years; provider B

The acceptability of a digital service was also discussed in relation to the COVID-19 pandemic, with participants more willing to engage with a DHI, having become more accustomed to doing things via technology during COVID-19–related restrictions, which made the NHS-digital-DPP more acceptable. Many participants also referenced the current strain on primary care as important in their decision to adopt the app, postulating that they would not be likely to be offered any other form of support for their risk because of the pressures on HCPs and primary care related to COVID-19. Many participants stated having accepted that digital delivery of health care would be the norm, going forward:

Let’s be very honest on that...there’s no choice. In today’s scenario, I think at least you have this option for you to do it, so you need to be beneficial about what you’re getting out of these programmes in the digital format. I think that’s the way we have to take it.

Participant 8; male; aged 45 years; provider A

In addition, because of COVID-19, several participants expressed continued fear of group interactions, and thus, having access to a digital service was viewed as critical for engagement in the NHS-DPP:

I think at the moment I would have possibly turned down the group, because I’m still conscious of COVID, I’m not too comfortable going into groups at the moment...I don’t want to risk getting ill again. And I just think that separation of having it online just works for me and makes me feel more comfortable.

Participant 25; female; aged 57 years; provider C

More generally, convenience and accessibility were the main features of the NHS-digital-DPP that appealed to people, with participants reporting that they could fit their use of the NHS-digital-DPP in around work patterns, caring responsibilities, and other daily activities. Being able to use the DHIs at a time that suited the participants helped them to feel in control of their use:

So it’s great to be able to do that just when I want to do it and that’s basically the reason. So I’m in control of what I do and when I do it.

Participant 12; male; aged 53 years; provider A

Other advantages of the NHS-digital-DPP included anonymity and privacy (participant 10)_._ For some participants, this was important as they felt stigma about being overweight and valued not having to interact with others in person:

I feel it’s different because it’s not at all – and actually it’s not about weight loss, there’s no clapping, it’s all secret, so we don’t really know [what people look like].

Participant 22; female; aged 58 years, provider D

Generally, the ability to use and engage with the DHIs was high. A few participants had experienced problems with initially downloading the apps but reported that these issues were resolved quickly with assistance from program providers. Even those who described themselves as having basic computer skills found the DHIs easy to engage with.

The features that participants reported most commonly engaging with were tracking features, including food, weight, and exercise physical activity tracking. Most participants (19/24, 79%) reported using these features daily, and the tracking aspects were conceptualized as the main part of the DHIs by many participants. Tracking features helped participants to feel accountable to the DHIs:

It’s accountability. I know what I need to do...I just don’t do it...I think for me it’s accountability. The weight metrics and the step metrics.

Participant 17; male; aged 44 years; provider D

However, there was variability across providers regarding the appraisal of the quality of features. Several participants from a provider were dissatisfied with the tracking abilities of the app and even described having to supplement the technologies with other apps that they had installed:

Yeah the app is extremely basic. I had my own other one which is a lot more detailed...so I’ll do that, that’s fine.

Participant 10; female; aged 65 years; provider A

Some participants commented that the *prediabetes* aspect of the DHIs were not always prominent, feeling that primarily this was a “weight loss” program. Many participants made the link between weight loss and risk reduction; however, for some participants, it was not clear why a weight loss program would reduce diabetes risk. Some were disappointed with the lack of content specifically about *prediabetes*:

Nobody’s really said to you, “OK, this is what type 2 diabetes is. This is how they [food points] relate to your blood sugar,” there’s no sort of X plus Y equals Z sort of thing.

Participant 6; male; aged 31 years; provider B

There’s no suggestions on how you should be making your diet better so that you don’t actually get diabetes.

Participant 13; female; aged 55 years; provider A

One of the advantages of the NHS-digital-DPP was the ability for tailoring. The health coaches were perceived to be the main mode of delivering tailored content, for example, by sending suitable articles to participants, delivering tailored information during conversations, and helping people to set individual goals:

I was quite reassured when I first got involved that it wasn’t going to be a one-size-fits-all type approach; it was going to be tailored to me...I’m finding it really quite useful, as reminders, and as I say, the information she’s provided, it’s being tailored.

Participant 27; female; aged 65 years; provider C

However, as discussed previously, not all participants believed that the degree of tailoring was sufficient. Much of the variability reported about the adequacy of tailoring came from expectations and perceptions of how good the health coaching features of the apps were:

And this is where things are similar that I’ve tried before, and I haven’t really done it because it feels very much like it’s one person managing 400 people and it’s do that which is generic response...they’re not cut and paste files, but at the same time I get the impression that she’s...told...you can’t say this, don’t talk about this kind of thing. So, it’s on brand in terms of their philosophy.

Participant 17; male; aged 44 years; provider D

#### Reflexive Monitoring

This theme relates to how participants appraised their use of the NHS-digital-DPP.

Most participants attributed their prediabetes diagnoses to being overweight. Therefore, most of them spoke about reducing weight as their desired outcome of using the DHIs. Those who were aiming to lose weight cited being motivated to continue use because they could observe changes in weight, for example, by noticing clothes feeling loose and decreasing weight measurements:

Already I’m a different shape, I have lost, I’ve lost eight pounds, and you know, every pound I lose, I can feel my clothes are better, can do my forward bends in yoga better, so you know, I know things are better and I’ve got more energy again, which is possibly this and the vitamin D together because they were, you know, that’s all success already.

Participant 22; female; aged 58 years; provider D

However, for some participants, especially those who were not overweight, their motivation for adopting the NHS-digital-DPP was to reduce their blood glucose levels and get out of the *prediabetic* range. For these participants, it was less clear how they could monitor their progress. Participants spoke about not knowing if or how they could obtain another blood glucose reading or how *prediabetes* and risk of diabetes would be monitored following diagnosis. This was seen as important for some participants to assess DHI efficacy and to maintain “motivation” for use (participant 20 and 21):

Another blood test would be quite good. If I...could see that it was having results, but it hadn’t got me all the way there yet, I would probably be very, really stick quite, you know, as rigidly as you can to it.

Participant 21; male; aged 43 years; provider D

Yes, I think, I mean, one of the things that I’m just curious about is I don’t know when I’d be getting another prediabetes check. And this is where the app doesn’t seem to be linking into the NHS procedures.

Participant 10; female; aged 65 years; provider A

Generally, those using the DHIs reported being motivated to continue use. Although participants had only been using the DHIs for few weeks, many reported increased feelings of efficacy toward preventing diabetes because of using the DHIs and observing changes:

So, the risk level or the probability...is not as significant as I thought initially when it started...it look like that at the minute at least with all the changes I could make through the programme duration, it might help to get it below the levels...to ensure it is under control.

Participant 8; male; aged 45 years; provider A

Others reported that over time, they anticipated learning enough from using the DHIs to allow them to embed changes into their lifestyle and thus would not need to continue engaging. This was also true for reducing use. People reported anticipating using the tracking features less frequently when they had learned enough or had reached a weight at which they were happy:

I’m imagining it [use] will get less and less. Obviously, that’s when I can manage it all myself. It’s just at the beginning, where you’re learning, again. So hopefully, I will be learning, myself, as I go, and I won’t need as much support, and it’ll gradually tail off, and it should just become my normal day’s living, really.

Participant 25; female; aged 57 years; provider C

However, as the NHS-digital-DPP was only being made available to participants for a fixed period of time (9 months), there were some concerns about how participants would continue the changes they had begun when their access ended:

I would like to continue with that because I just would worry that if I didn’t use it, I would go back to the way I was. So I will continue to use it.

Participant 3; female; aged 57 years; remote group

## Discussion

### Principal Findings

DHIs for diabetes prevention are showing considerable potential for behavior change among those who engage with them. However, less is known about the factors that influence uptake and engagement with these interventions in real-world populations. To promote participation in and to inform the development of future digital-DPPs, more evidence about these critical factors is needed. This study explored decision-making around uptake of and engagement with the NHS-digital-DPP, with participants who had been offered referral as part of routine NHS care. The views of those who did and did not take up the NHS-digital-DPP and those who chose a different delivery mode are represented.

The findings related to both the NHS-DPP generally and the NHS-digital-DPP specifically. Psychological factors related to beliefs about vulnerability to diabetes, self-efficacy for reducing risk, and self-identify and implementation factors including issues with referrals and lack of engagement from HCPs were barriers to the uptake of the NHS-DPP. Factors that related specifically to the uptake of a digital service included perceptions about usefulness in supporting behavioral modifications, perceptions about accessibility and convenience, and views about participating in a group. Specific features of the DHIs including health coaches and tracking features that promoted accountability and motivation were important for promoting engagement.

Many participants perceived the NHS-digital-DPP as an acceptable service to help reduce the risk of developing T2DM, and many reported having been supported to make behavior changes. The benefits of the NHS-digital-DPP were often discussed in contrast to perceptions of face-to-face and group-based services, highlighting the need for a range of delivery options for diabetes prevention to ensure that participants can access a service that meets their specific needs and preferences and thus promote engagement.

### Strengths and Limitations

One of the main strengths of this study is the collection of data from a real-world population including those who accepted the NHS-digital-DPP, those who opted for a different delivery mode, and those who did not take up the offer. Studying the reasons for not taking up digital interventions is essential for overcoming proinnovation bias [48]. However, it is likely that given the small number of participants in this subgroup, there are still factors that remain obscured, which could be explored with further studies into nonuptake. Furthermore, despite best efforts, because of the way in which the NHS-digital-DPP is offered to participants (usually not until participants have made contact with providers), it remains difficult to isolate views on nonuptake that relate specifically to the digital delivery mode, as opposed to the NHS-DPP more broadly.

Although the sample was diverse in terms of ethnicity, age, and sex, there was less diversity represented in terms of other characteristics including socioeconomic status, education, digital access, and computer skills. Thus, these findings are unlikely to fully represent the experiences of those on the other side of the so-called digital divide, which represents inequalities in accessing and using digital technologies [49]. For example, previous studies have shown that adoption of DHIs may be less among those with low socioeconomic status, [50], less computer experience [51], and less access to social networks [52]. Engagement with and adherence to DHIs may also be less among those with low education levels and socioeconomic status [53]. Future studies with individuals from these groups may highlight additional findings about uptake of and engagement with digital-DPPs.

The primary focus of the study was on factors that influenced uptake and initial engagement decisions, and thus, participants had access to the DHIs for a relatively short time frame. Ongoing research by this team is examining users’ patterns of use of the NHS-digital-DPP, which will provide findings on how the DHIs are used longitudinally. Further qualitative studies to assess long-term experiences with the NHS-digital-DPP, especially around decisions to continue or cease use, would complement this ongoing study.

### Comparisons With Previous Literature

Participants often contrasted themselves with people that they thought were typical of a diagnosis of diabetes and highlighted ways in which they were different (not overweight, more active, and more knowledgeable). These participants found it difficult to identify themselves as being in an “at-risk state” [4], as this conflicted with their own perceptions of having a healthy lifestyle creating a distance to future risk. Similar findings were reported in our qualitative study with NHS-DPP participants, which found that people with prediabetes resist the notion that they are “candidates” for diabetes as this contradicted their perceived identity as *healthy* individuals [54]. In this study, not feeling typical of someone who develops diabetes also led participants to question how much they would benefit from the NHS-digital-DPP.

Findings emphasized the importance of several constructs of the HBM [43] including perceived susceptibility and severity, cues to action, and self-efficacy. Many participants were shocked by their diagnosis of prediabetes, and their understanding about the diagnosis was mixed. This was often related to the way in which the diagnosis had been delivered, often via letter or SMS text message with limited information. However, most participants reported feeling strongly that if they did not take preventive action, they would be susceptible to developing diabetes. However, there were mixed views about how severe diabetes may be if it developed, which were mediated by the way in which participants had been informed about their risk by their HCP and personal experiences with diabetes. A meta-analysis of barriers to and facilitators of lifestyle changes in people with prediabetes identified the point at which people become aware of being at high risk of developing T2DM and realize the potential threat to their health as a vital facilitator of healthy lifestyle changes [35], which were findings supported by a meta-analysis exploring risk appraisals that showed altered risk perceptions have impact on intentions to change behavior and on changes in behavior itself [55]. In many cases, there seemed to be a missed opportunity to engage participants with their diagnosis and preventable actions because of the way in which this information was communicated to participants. Several previous studies have highlighted that the participant’s assessment of the seriousness of prediabetes may be influenced by HCP’s communication and behavior [21,34,56].

Furthermore, health professionals’ communication around the NHS-DPP was perceived as a critical cue to action, that is, the stimulus needed to trigger the decision-making process to accept a recommended health action. In cases where participants had not taken up the NHS-digital-DPP, this was because of poor referrals, including lack of information about the digital option or offers not being received. Those who had taken up the NHS-digital-DPP reported being unclear about the program’s aims and content, because this had not been communicated adequately at the point of referral. A recent study examining the US National diabetes prevention program also found that cues to action were determinants of enrollment, specifically that clear information about the diagnosis of prediabetes and decision support for joining a lifestyle intervention, especially from a trusted health care provider, were critical [45]. However, improving communication around referrals by HCPs may be exacting. For example, a previous study that examined HCPs’ views of a digital T2DM self-management program highlighted the difficulties of HCPs in absorbing the additional tasks needed to provide adequate referrals to the program into an already overwhelming workload [57], and several other studies have identified resource constraints as barriers to HCPs referring to digital services [58,59].

Self-efficacy for being able to reduce the risk of diabetes was generally high among participants, with many viewing a diagnosis of prediabetes as an opportunity to make healthy lifestyle modifications, and self-efficacy for behavior changes improved after participants started using the NHS-digital-DPP. As with previous studies [35], having former positive experiences with exercise and diet facilitated self-confidence for engaging in these behaviors. Participants’ self-confidence for behavior changes was bolstered by positive feedback, for example, seeing weight loss and feeling healthy as a result of using the NHS-digital-DPP. Features of the DHIs, which helped participants track and visualize their progression toward goal attainment, had a positive impact on perceptions of being able to prevent diabetes. However, participants were disappointed at the lack of feedback on whether behavior changes were having an impact on risk of developing T2DM.

There were participants with a family history of diabetes who were more likely to perceive disease progression as an inevitability. Previous studies have emphasized the importance of understanding social constructs including *inevitable* social norms related to genetic predispositions to diabetes, which can influence decisions to engage with health care advice and implement behavior changes [60].

Proactive health coaching was appraised positively by most participants and was important for their decisions to take up and maintain engagement with the program. Health coaching helped participants to access relevant information, set personal goals, review progress, tailor the DHIs, and provide *human* contact. Previous studies of digital-DPP interventions have shown that health coaches are valued by users [61], may enhance participation and engagement [62], and may enhance the efficacy of digital-DPP interventions on weight loss [21]. Input from professionals may foster feelings of accountability and a sense of being monitored, which have been shown to facilitate lifestyle changes [35].

Despite an emerging body of literature suggesting that peer support is important for engagement with digital diabetes prevention and outcomes [24,35,63], this study found mixed views regarding participants’ desire for peer support. Those who had opted for remote groups did so because they wanted to interact with others. However, among those who opted for the digital service, many reported not wanting or needing to engage with peers, and those who engaged with peers reported that the peer support features were underused by other users, thus decreasing their motivation to engage with these aspects.

### Implications

It is likely that adequate discussion by HCPs about prediabetes and T2DM risk would increase patients’ knowledge about disease severity and emphasize the preventable nature of T2DM, thus increasing self-efficacy for behavior changes. The findings also suggest that better communication may raise awareness about the digital service, provide endorsement, and help to sustain participant engagement. Future studies could focus on HCPs’ perceptions of these factors to identify barriers to referral. For example, recent study by this team on the implementation of the NHS-DPP suggests that individuals responsible for the local commissioning and implementation of NHS-DPP report having minimum knowledge about the NHS-digital-DPP in terms of the content [64]. Therefore, strategies to promote patient uptake could focus on raising awareness about the NHS-digital-DPP among those responsible for referring to it. Thought could also be given to ways to ensure that patients receive adequate information that does not have an impact on HCP resources, for example, through direct-to-patient marketing of the NHS-digital-DPP or peer-led information sessions. In addition, HCPs could be provided with specific tailored materials to provide to certain groups. For example, specific messaging for those with a genetic diabetes risk or for those who are not overweight may promote the value of behavior changes and NHS-DPP.

Digital-DPPs are still in their infancy, and it is not yet clear how best to optimize their delivery to enhance the desired clinical outcomes. However, findings from this study suggest that accountability and monitoring affected the participants’ early experiences and encouraged uptake and engagement. Accountability was frequently described in terms of interactions with the health coaches. Motivation for continued use was driven by the ability to monitor progress. The DHIs had specific features to help participants visualize and monitor goals that were perceived as motivating. Thus, these findings suggest that digital-DPP interventions should feature elements of accountability and automated monitoring systems. Furthermore, it is important to consider how users of digital-DPPs who are not overweight can be supported to monitor their progress. For example, future implementation efforts could make it clear to participants about how and when they can repeat blood tests to monitor diabetes risk, and a blood test could be potentially offered as part of the service.

Finally, thought could be given to tailoring digital-DPP interventions based on participants’ desire to interact with others, for example, placing those with a desire for peer support together as a group.

### Conclusions

This study provides important findings on factors that influence decisions to take up and engage with the NHS-digital-DPP. Findings suggest that participants found the DHIs to be convenient, accessible, and useful in supporting behavior changes. Specific features including health coaches and tracking tools were important for initial motivation and accountability. The study also highlights the importance of communication about diabetes risk and NHS-digital-DPP.

## Appendix

Multimedia Appendix 1 Topic guide.

Multimedia Appendix 2 Summary of factors important for uptake of and engagement with National Health Service Healthier You: Diabetes Prevention Programme (NHS-DPP; generally) and National Health Service Healthier You: Digital Diabetes Prevention Programme (NHS-digital-DPP; specifically).

## Abbreviations

DHI: digital health intervention
digital-DPP: digital diabetes prevention program
f2f-DPP: face-to-face diabetes prevention program
HBM: health belief model
HCP: health care professional
NHS: National Health Service
NHS-digital-DPP: National Health Service Healthier You: Digital Diabetes Prevention Programme
NHS-DPP: National Health Service Healthier You: Diabetes Prevention Programme
NHS-f2f-DPP: face-to-face version of the National Health Service Healthier You: Diabetes Prevention Programme
T2DM: type 2 diabetes mellitus
