# Supplementary material for: Influences on Patient Uptake of and Engagement With the National Health Service Digital Diabetes Prevention Programme: Qualitative Interview Study
Source: J Med Internet Res. 2023 Feb 28;25:e40961. doi: 10.2196/40961 (PMC10015356; doi:10.2196/40961)
Supplement: Multimedia Appendix 1 [file jmir_v25i1e40961_app1.docx]

**Supplementary material 1**

**Interview topic guide**

| Domain | Description | Primary questions |
| --- | --- | --- |
| Questions related to the Health Belief Model | | |
| Perceived susceptibility | Beliefs about the risks of pre-diabetes and type 2 diabetes | How did being told you are at risk of developing diabetes feel to you?  How likely do you feel it is that you will develop diabetes? |
| Perceived severity | Beliefs about the seriousness of type 2 diabetes and its consequences | What did you understand by pre-diabetes/ at risk of diabetes?  If you were to develop diabetes, what impact do you think this might have on you / your way of life?  Is there a history of diabetes in your family? |
| Perceived benefits | Beliefs about the effectiveness of taking action to reduce risk of seriousness | What do you think you might need to do to prevent diabetes developing?  How do you feel about making lifestyle changes that might reduce your risk of diabetes?  How (if at all) did you think a diabetes prevention programme might help you?  Who do you think programmes such as “healthier you” are aimed at?  *For those who accepted offer of NHS-DPP*  From the information you received can you describe what you expected from the “healthier you” programme?  What factors did you consider when choosing to start the programme?  *For those who declined offer of the NHS-DPP*  Do you have any plans to take any actions in relation to your health and lifestyle?  Are there any factors that might motivate you to make changes to prevent diabetes? |
| Perceived barriers | Beliefs about the obstacles to behaviour change | Are there any factors that might prevent you to make changes to prevent diabetes?  How did you think the “healthier you” programme might fit (or not) into your everyday life?  Can you describe any pros and cons to participating in a programme like “healthier you”?  *For those who accepted offer of the NHS-DPP*  Are there any factors that might prevent you from using the programme?  *For those who declined offer of the NHS-DPP*  What factors did you consider when choosing to decline the offer of the programme? |
| Cues to action | Factors that activate “readiness to change” | How were you first made aware that you might be at risk of developing diabetes?  What do you remember discussing with your GP/Nurse about blood sugar levels?  When you were told that you might be at risk of developing diabetes, what information or support, (if any) were you offered?  How was the “healthier you” programme described to you by the person who offered it to you/referred you?  How do you feel about digital programmes as a way to help prevent people developing diabetes?  Are there other things that would have been useful for you to know about the “healthier you” programme? |
| Questions relating to uptake and engagement with the NHS-digital-DPP | | |
| Choice of service |  | Were you given information about the different ways the “healthier you” programme is delivered?  Could you tell me a little about your decision making when choosing which version of the “healthier you” programme to start?  What appealed to you about the version you opted for? |
| Uptake |  | Can you briefly talk me through the process of signing up to the “healthier you” programme?  Are there other things that would have been useful for you to know/have when you joined the programme? |
| Engagement |  | Can you tell me how you have been using the programme so far?  How motivated do you feel to continue using this programme and why?  How do you feel having started this programme?  Have you made any changes to your lifestyle since using this programme?  Do you have any recommendations about how this programme could be improved in terms of the way you initially find out about it and the joining up process?  Could I ask how you would describe the programme to a friend or family member in a sentence or two |
