# Supplementary material for: Influences on Patient Uptake of and Engagement With the National Health Service Digital Diabetes Prevention Programme: Qualitative Interview Study
Source: J Med Internet Res. 2023 Feb 28;25:e40961. doi: 10.2196/40961 (PMC10015356; doi:10.2196/40961)
Supplement: Multimedia Appendix 2 [file jmir_v25i1e40961_app2.docx]

Multimedia Appendix **2.**

Summary of factors important for uptake of and engagement with NHS-DPP^a^ (generally) and NHS-digital-DPP^b^ (specifically).

| Themes and categories | | NHS-DPP | | NHS-digital-DPP | | |
| --- | --- | --- | --- | --- | --- | --- |
|  | | Factor | Description | Factor | Description | |
|  | | | | | | |
| **Knowledge and understanding** | | | | | | |
|  | **Uptake** | | | | | |
|  |  | Diagnosis delivery and understanding | The way an HCP^c^ delivers the diagnosis and how well the patient understands the diagnosis influences their decision to take up the NHS-DPP | —^d^ | — | |
|  |  | HCP communication about prediabetes, diabetes risk, and NHS-DPP | The style of communication, explanations of disease and risk, and descriptions of the NHS-DPP influenced decisions to take up the NHS-DPP | — | | — |
| **Referral process** | | | | | | |
|  | **Uptake** | | | | | |
|  |  | Referral mode | Conversations around the offer were viewed as more positive than participants receiving letters | Provision of information about digital option | Lack of knowledge about the digital option was a barrier to uptake | |
|  |  | HCP communication and follow-up | HCP follow-up about whether participants had taken up the referral was viewed positively and had an impact on the decisions to take up | — | | — |
|  |  | Delays with gaining access to program | Frustration about delays in getting started with the program influenced perceptions of the program; for some people, this involved considerable work to get access | — | | — |
| **Self-efficacy** | | | | | | |
|  | **Uptake** | | | | | |
|  |  | Previous experiences | Previous experiences with behavioral modification had an impact on self-efficacy for making further changes | Experience with digital technologies | Previous technology use has an impact on self-efficacy for NHS-digital-DPP use | |
|  |  | Genetic risk | Genetic risk factors for T2DM^e^ had an impact on self-efficacy for reducing risk via behavioral modifications | — | | — |
| **Self-identity** | | | | | | |
|  | **Uptake** | | | | | |
|  |  | Self-identity | Whether the individual identifies as part of the target population influenced perceptions of program’s relevance and suitability | Self-identity | Perception of suitability of a digital service related to participants’ sense of self | |
| **Motivation and support** | | | | | | |
|  | **Uptake** | | | | | |
|  |  | Peer support | Peer support was important for those opting for remote delivery over digital | Peer support | Not wanting to interact in groups was important for deciding to opt for the NHS-digital-DPP over group-based format | |
|  | **Engagement** | | | | | |
|  |  | — | — | Professional support | Proactive health coaches were important for motivation and accountability | |
| **Advantages of digital service** | | | | | | |
|  | **Uptake** | | | | | |
|  |  | — | — | Convenience and accessibility | Perceptions of how well the use of the NHS-digital-DPP would fit into existing schedules and routines promoted uptake of the digital option | |
|  | **Engagement** | | | | | |
|  |  | — | — | Digital features | Features including tracking tools, tailoring, and health coaches promoted engagement with the NHS-digital-DPP | |
| **Reflexive monitoring** | | | | | | |
|  | **Engagement** | | | | | |
|  |  | — | — | Measuring progress | Observing and measuring progress was important for continued engagement | |

^a^NHS-DPP: National Health Service *Healthier You*: Diabetes Prevention Programme.

^b^NHS-digital-DPP: National Health Service *Healthier You*: Digital Diabetes Prevention Programme.

^c^HCP: health care professional.

^d^Theme is either related to the NHS-DPP broadly or the NHS-digital-DPP specifically, not both.

^e^T2DM: type 2 diabetes mellitus.
